# 3D surface reconstruction of the femur and tibia from parallel 2D contours

**DOI:** 10.1186/s13018-022-02994-w

**Published:** 2022-03-05

**Authors:** Bigui Lin, Dadi Jin, Miguel Angel Socorro Borges

**Affiliations:** 1 Graduate School of Southern Medical University, Guangzhou City, 510515 China; 2 Orthopedics Department of the Third Affiliated, Hospital of Southern Medical University, Guangzhou City, 510630 China; 3 Sovajo Medical Tech Limited Company, Hangzhou, 311122 China

**Keywords:** Area optimization, Surface reconstruction, Surface triangulation

## Abstract

**Background:**

Segmented structures, such as bones, are typically stored as 2D contours contained on evenly spaced images (slices). Contour interpolation algorithms to turn 2D contours into a 3D surface may differ in their results, causing discrepancies in analysis. This study aimed to create an accurate and consistent algorithm for the interpolation of femur and tibial contours that can be used in computer-assisted surgical navigation systems.

**Methods:**

The implemented algorithm performs contour interpolation in a step-by-step manner, determining an optimal surface between each pair of consecutive contours. Determining such a surface is reduced to the problem of finding certain minimum-cost cycles in a directed toroidal graph. The algorithm assumes that the contours are ordered. The first step in the algorithm is the determination of branching patterns, followed by the removal of keyholes from contours, optimization of a target function based on the surface area, and mesh triangulation based on the optimization results and mesh seal.

**Results:**

The algorithm was tested on contours segmented on computed tomography images from femoral and tibial specimens; it was able to generate qualitatively good 3D meshes from the set of 2D contours for all the tested examples.

**Conclusion:**

The contour interpolation algorithm proved to be quite effective using optimization based on minimizing the area of the triangles that form the 3D surface. The algorithm can be used for the 3D reconstruction of other types of 2D cuts, but special attention must be paid with the branches, since the proposed algorithm is not designed for complex branching structures.

## Introduction

Total knee arthroplasty (TKA) can be performed via surgery using computer-assisted surgical navigation systems, with the goal of improving surgical accuracy and precision. These types of surgeries need to register the patient’s anatomy in the application; after bone segmentation, an accurate bone model surface must be generated. Segmented bones are typically stored as 2D contours contained on evenly spaced images (slices). Contour-interpolation algorithms turn 2D contours into a 3D surface; however, the results can differ between algorithms, causing discrepancies in analysis [1]. This study’s goal was to create an accurate and consistent contour interpolation algorithm that can generate a good 3D surface for use during computer-assisted TKA. The process of generating a 3D model or surface from a set of parallel slices is a difficult process widely discussed in the scientific literature; this process is associated with several problems, as follows [2]:

- The correspondence problem involves deciding which contours from two different sections should be linked together in the generated surface. This is solved by determining the topological adjacency relationships between the contours of a set of slices. A solution to the correspondence problem determines the coarse topology of the final surface.
- The branching problem arises when an object is represented by a different number of contours in adjacent sections, in which case the standard method for solving the tiling problem cannot be used directly. A solution to the tiling and branching problems determines the topology of the surface and its coarse geometry.
- The keyhole problem arises when there are non-adjacent points that are very close to each other. This is solved by closing the keyhole.
- The surface-fitting problem involves fitting the “best” surface to the mesh computed by solving the above-mentioned problems. A solution to the surface-fitting problem produces a detailed description of the geometry of the reconstructed surface.
- The tiling problem consists of generating the “best” topological adjacency relationships between the points on pairs of contours from adjacent slices by constructing a triangular mesh from their points. A commonly chosen metric for determining the “best” topological adjacency is the minimization of the resulting surface area.
- The seal mesh problem arises after the triangulation is complete, because some gaps remain in the surface model; these gaps need to be sealed. These gaps in the surface are caused by the contours that reside on the top and bottom of the 3D model.

For several years, different solutions to these problems have been proposed in general; some of the main solutions are as follows:

Keppel [3] first minimized the problem of matching points in successive contours to a search problem on a toroidal graph; he used a metric based on enclosed volume and investigated heuristic methods for dealing with the problems associated with this metric. The major problems with this work were associated with the choice of “Maximize Volume” as the objective function to be optimized.

Fuchs et al. [4] provided an extensive analysis of the search problem and developed an efficient search method. They formalized Keppel’s approach and applied a divide-and-conquer technique to speed the search. They used minimal surface area as an example metric, avoiding many of the problems associated with Maximize Volume. The former is intuitively appealing and probably as good as any metric. However, this solution does not consider the keyhole problem.

Christiansen and Sederberg [5] described a greedy method based on “Minimizing Span Length.” Their method incorporated the normalization of size and position and was extended to handle branching structures. The method fails in some fairly common cases [6].

Cook [7] used a greedy algorithm to produce a surface with a metric based on direction vectors from centroids to individual points. The application of this technique is relevant to medical imaging; it is intuitively appealing for convex objects, but there are problems with severely concave objects.

Ganapathy and Dennehy [8] introduced a new heuristic, based on “Normalized Arc Length.” Their immediate application was in the ultrasonic detection of flaws in pressure vessels. The nature of their heuristic virtually required that they use a greedy search algorithm. The metric and algorithm were well matched. Their approach traded correctness for speed, apparently to good effect. However, this method produced unacceptable tilings for rather common situations [6].

Sunderland [1] used a triangulation algorithm based on the minimization of the length of edges spanning the contours via dynamic programming. The algorithm was tested on contours segmented on computed tomography (CT) images. The algorithm was able to appropriately handle individual occurrences of the issues of rapid changes in shape and branching and keyhole contours; however, the presence of several of these issues in the same location simultaneously was found to cause problems for the final surface mesh.

Moriconi [9] combined information extracted from both voxel image segmentation and implicit surface-streaming methods employed in computer graphics. This was done by first extracting a dense cluster of oriented points from the binary segmentation of the organ and then streaming the 3D surface from the oriented points using a wavelet-based reconstruction algorithm. This algorithm showed poor image quality in cases where there were several rapid changes in shape.

Solutions to the problem associated with pairs of contours with a similar shape are handled well by the methods discussed above. When the contours have dissimilar shapes or similar shapes oriented differently, most or all of these methods, especially those not based on an optimization process, fail to produce acceptable results.

The algorithm proposed in this work seeks to find a suitable solution to the 3D reconstruction of the femur and tibia; this algorithm is based on an area minimization approach.

## Methods

**Fig. 1:**
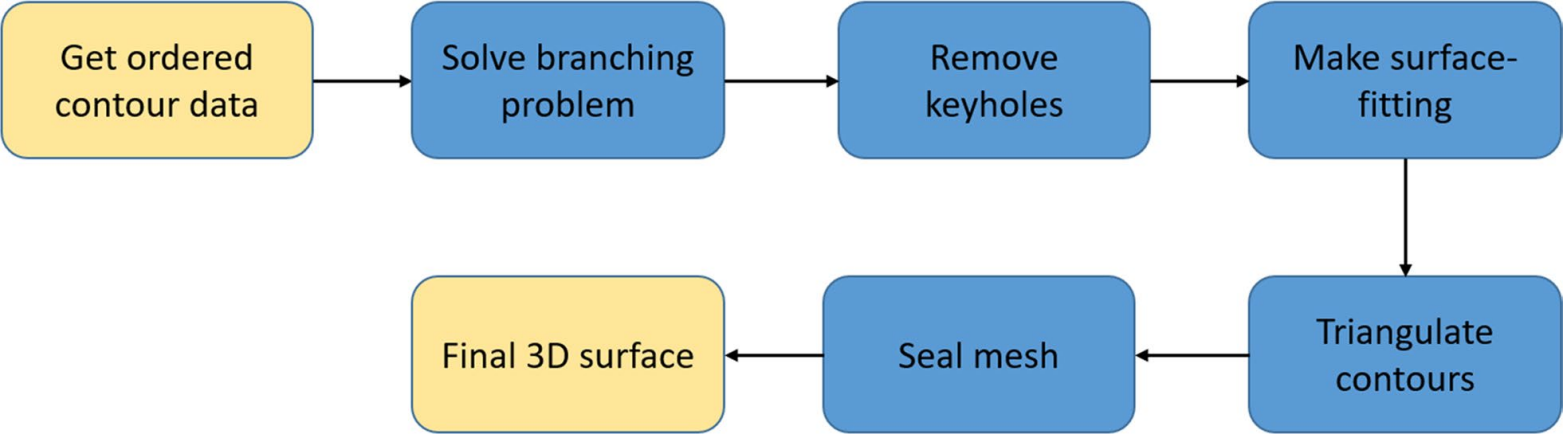
Data flow diagram. The process of converting a set of ordered 2D contours into a corresponding surface mesh

The algorithm described herein was designed with the intent to preserve the original points and to avoid introducing new points. There is only one case in which this rule is not fulfilled, and this is during the keyhole handling process, because the points need to be removed from within the channel. The 3D mesh construction procedure is shown in the data flow diagram in Fig. 1.

The algorithm performs the contour interpolation in a step-by-step manner, determining an optimal surface between each pair of consecutive contours. These surfaces can be constructed from elementary triangular tiles, each defined between two consecutive points on the same contour and a single point on an adjacent contour [4]. Determining such a surface is reduced to the problem of identifying certain minimum-cost cycles in a directed toroidal graph. This algorithm assumes that the contours are ordered. Each of the processes described below are applied for each pair of consecutive contours, except for the last process (seal mesh), which is performed when all the contours have been triangulated.

### Branching

**Fig. 2:**
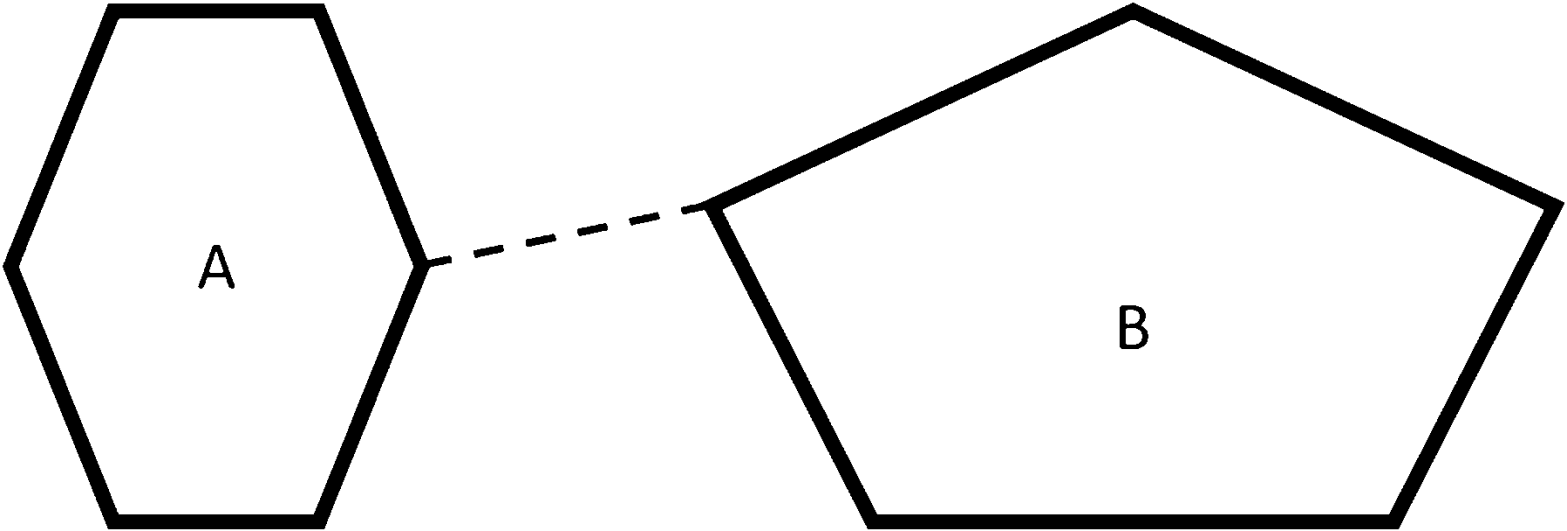
A simple case of the branching problem. Two contours in one slice merge into one contour in an adjacent slice using an edge between the nearest points

An instance of the branching problem exists when a local area of an object represented by *m* contours in one section is represented by *n* contours in an adjacent section where *m* ≠ *n* and *n* > 0 [6]. To solve this problem, this study uses a method that will handle non-complex branching structures. The key idea is joining the sections of the contours with branching problems, adding one edge between the nearest points, as shown in Fig. 2.

### Keyholes

The existence of keyholes within contours can cause problems with the process of triangulation, as they can cause the triangulation algorithm to incorrectly connect to points contained within the channel. To remove the keyholes from the contours, each contour is addressed individually in a pre-processing step, comparing each point in the contour to all other points in the same contour. If two points within a contour are within a specified threshold distance from each other and not considered to be adjacent to each other, these two points are noted to be in conflict with each other [1]. Once all the points in the current contour have been assessed, the algorithm walks through the list of points for the current contour, rebuilding the contour and removing the points inside keyholes and adding additional edges as is necessary to remove the keyholes.

### Surface fitting and triangulate contours

**Fig. 3:**
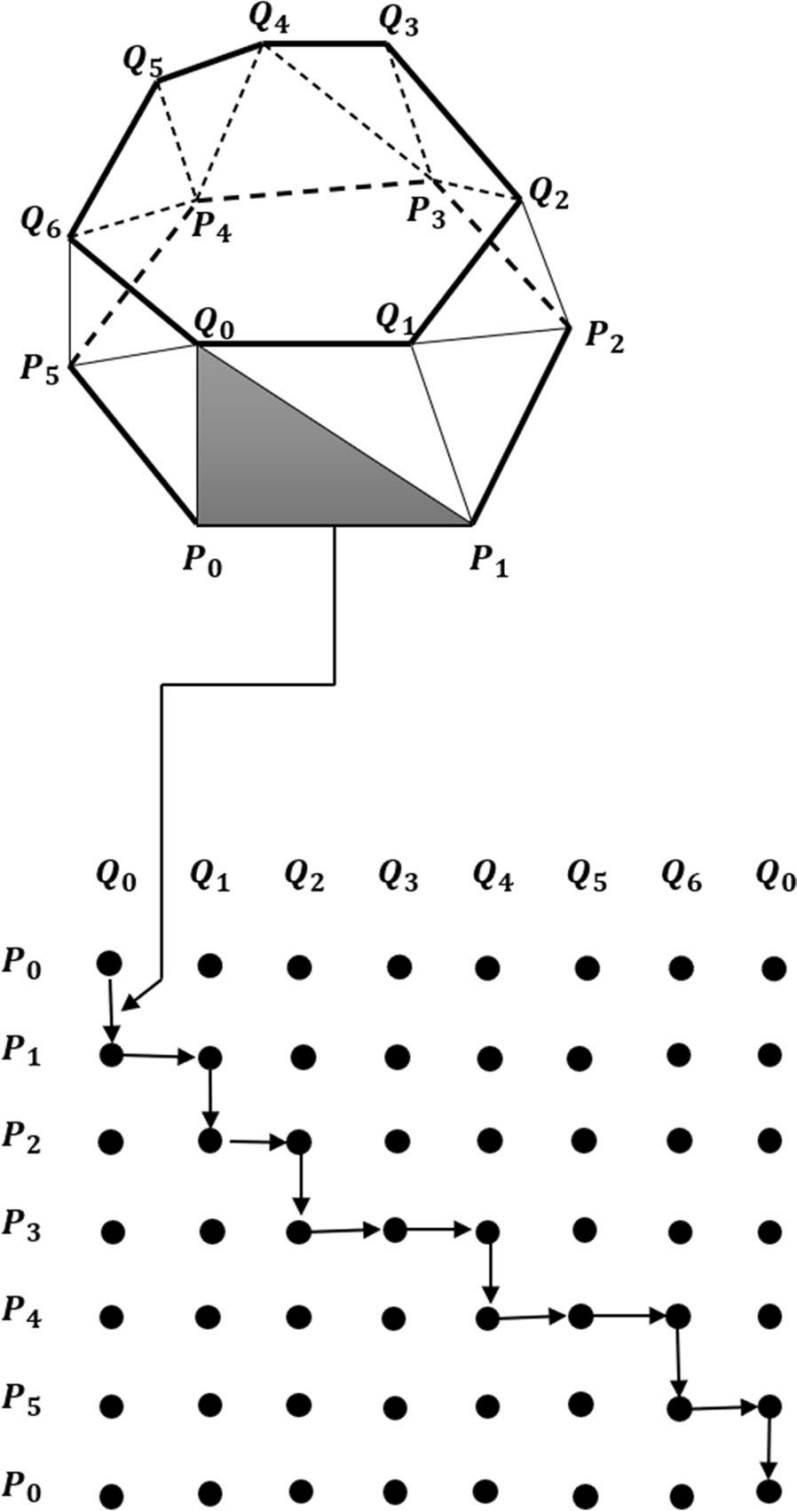
Tiling process. The tiling process is performed by finding a minimum-cost cycle in the graph; as a result, a triangulated surface is obtained. Each edge of the graph represents a triangle on the surface. The weight of each edge is the area of the triangle it represents

The main goal of triangulating contours (tiling) and solving the surface-fitting problem is finding the best correspondence between points in successive contours. This problem can be reduced to a search problem on a toroidal graph, as demonstrated in a previous study [3]; in the aforementioned study, contours are represented by ordered lists of data points. Edges connecting neighboring points within the same contour are called Contour Segments. Edges connecting a point from one contour to a point from the other contour are called Spans. Spans are represented by nodes in the graph. Tiles are represented by arcs between nodes in the graph. Tiling is found by finding a minimal-cost cycle in the graph, as shown in Fig. 3. Both problems are severely constraining, and many surfaces could solve them [10]. To choose one correct surface, it is necessary to perform an optimization process with respect to an objective function [11]. The chosen objective function should capture some notions of what a good surface is and should be easy to compute. Previous studies have reported the use of different metric functions to distinguish good surfaces from bad; some of the best-known metric functions are as follows:

- Maximize Volume: It is an obvious metric only for convex objects but is difficult to use. It calculates the contribution of a single tile to an objective function related to the total volume of an object.
- Minimize Area: It is a good metric. However, special care must be taken to handle certain cases with complex geometries or very distant slices, for example, in cases where there are two identical circular cross sections positioned in parallel planes, with their centers offset one diameter apart. Some of the metrics listed above will produce something like a double cone joined at a line, rather than the obvious skewed cylinder. The solution to this problem is to normalize the two cross sections such that their centers lie on an axis perpendicular to the planes of section [12]. The Minimize Area method is easy to compute and can be improved using normalized contours for the position and radius [2]; this is our metric of choice.

**Fig. 4:**
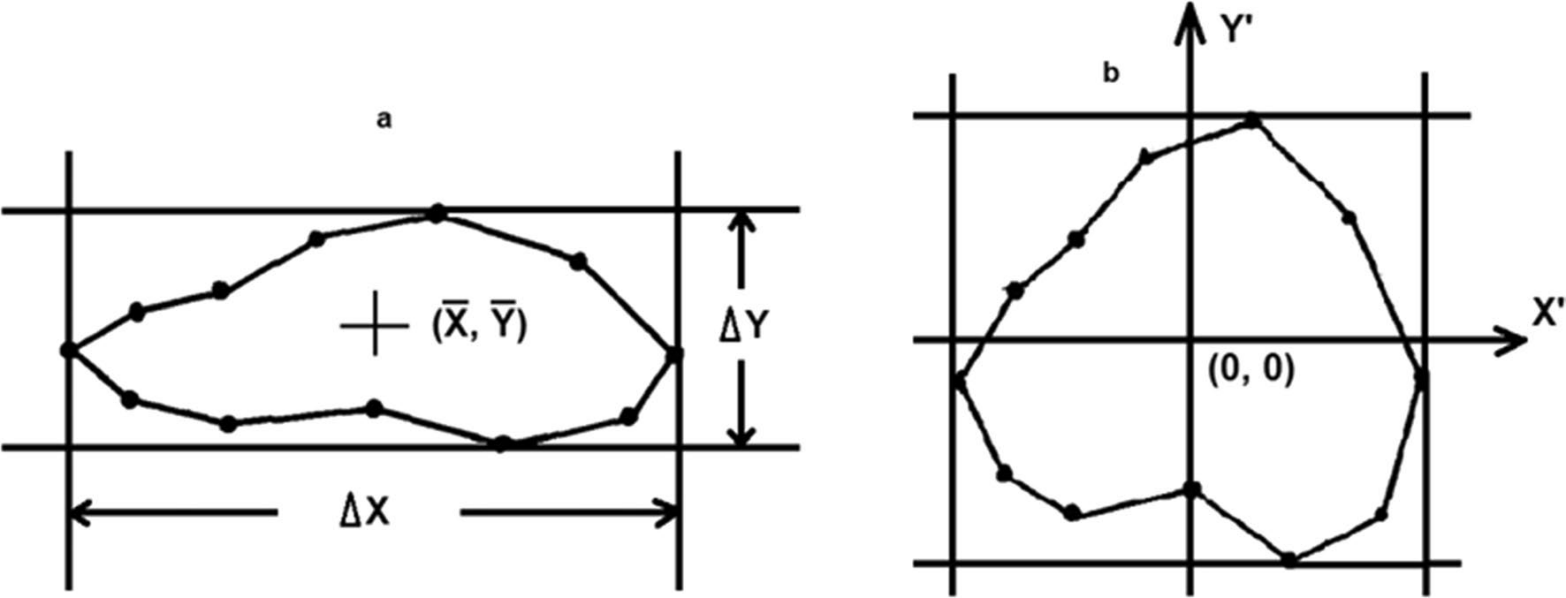
Contour normalization. **a** Original contour with its rectangular window. **b** Normalized contour

To normalize each slice with respect to its position, the midpoint of the contour is calculated in order to move the contour to the coordinate (0,0) as the center. For normalization with respect to its radius, a rectangular window that encloses the contour will be defined; using the length and width of this window, the contour coordinates are scaled according to a previous study [5], as shown in Fig. 4 and below:

In the above equation,  and  are the new coordinates. Since the objective function to be used is a function that minimizes the surface area, the total sum of the area of all the triangles generated on the surface must be minimized. Given three possible points, A, B, and C, to define a triangle, the area is calculated using the magnitude of the cross product of two vectors defined by these points as follows:

$$\overrightarrow {BA} = A - B$$

$$\overrightarrow {BC} = C - B$$

$$\vec{N} = \overrightarrow {BA} \times \overrightarrow {BC}$$

$${\text{Area}} = \frac{{\left( {\vec{N} \cdot \vec{N}} \right)^{\frac{1}{2}} }}{2}$$

**Fig. 5:**
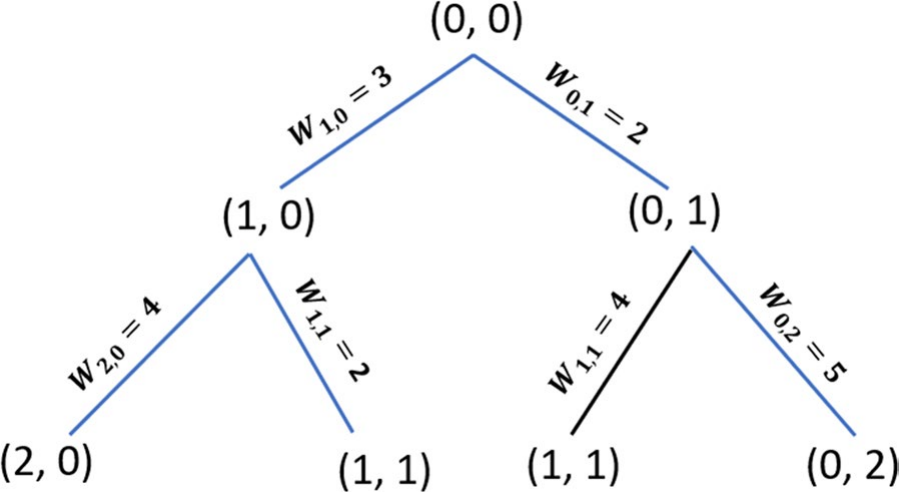
Possible paths. The right paths are indicated in blue. *W*(*i,j*) is the edge weight example that represents the triangle area

To start the tiling algorithm, the two closest points between each pair of consecutive slices are searched; these closest points will be the starting points of the triangulation and the first node of the graph. The weight (W) of each edge is given by the area of the triangle it represents; the minimum path from the first node $P_{{\left( {0,{ }0} \right)}}$ to the last node of the graph $P_{{\left( {n,{ }m} \right)}}$, where *n* is the total number of points of one of the consecutive contours and *m* is the total number of points of the other contour, is the solution that is sought. This minimal path guides how the triangulation between the two consecutive slices can be made. Due to the characteristics of the graph from a node $P_{{\left( {i,{ }j} \right)}}$, it is only possible to advance to the nodes $P_{{\left( {i + 1,{ }j} \right)}}$ and $P_{{\left( {i,{ }j + 1} \right)}}$; thus, all possible paths are generated. Each generated edge will contain a weight associated with the area of the triangle it represents. When repeating nodes are found, only the node that was reached by the shortest path will be taken; the others will be discarded (Fig. 5). As can be seen in Fig. 5, node (1, 1) can be reached in two different ways: through a path A [(0, 0) −> (1, 0) −> (1, 1)], with a weight of $W_{{\left( {1,{ }0} \right)}} + W_{{\left( {1,{ }1} \right)}} = 5$ and through a path B [(0, 0) −> (0, 1) −> (1, 1)], with a weight of $W_{{\left( {0,{ }1} \right)}} + W_{{\left( {1,{ }1} \right)}} = 6$. Because path A is shorter, path B can be discarded. The algorithm ends when the node $P_{{\left( {n,{ }m} \right)}}$ is reached. The shortest path that leads to node $P_{{\left( {n,{ }m} \right)}}$ is the path sought and the solution to the triangulation problem between the two contours.

### Seal mesh

The gaps in the surface caused by the contours that reside on the top and bottom of the 3D model should be closed. For this case, it is enough to use a 2D triangulation algorithm, such as the Delaunay triangulation.

## Results

**Fig. 6:**
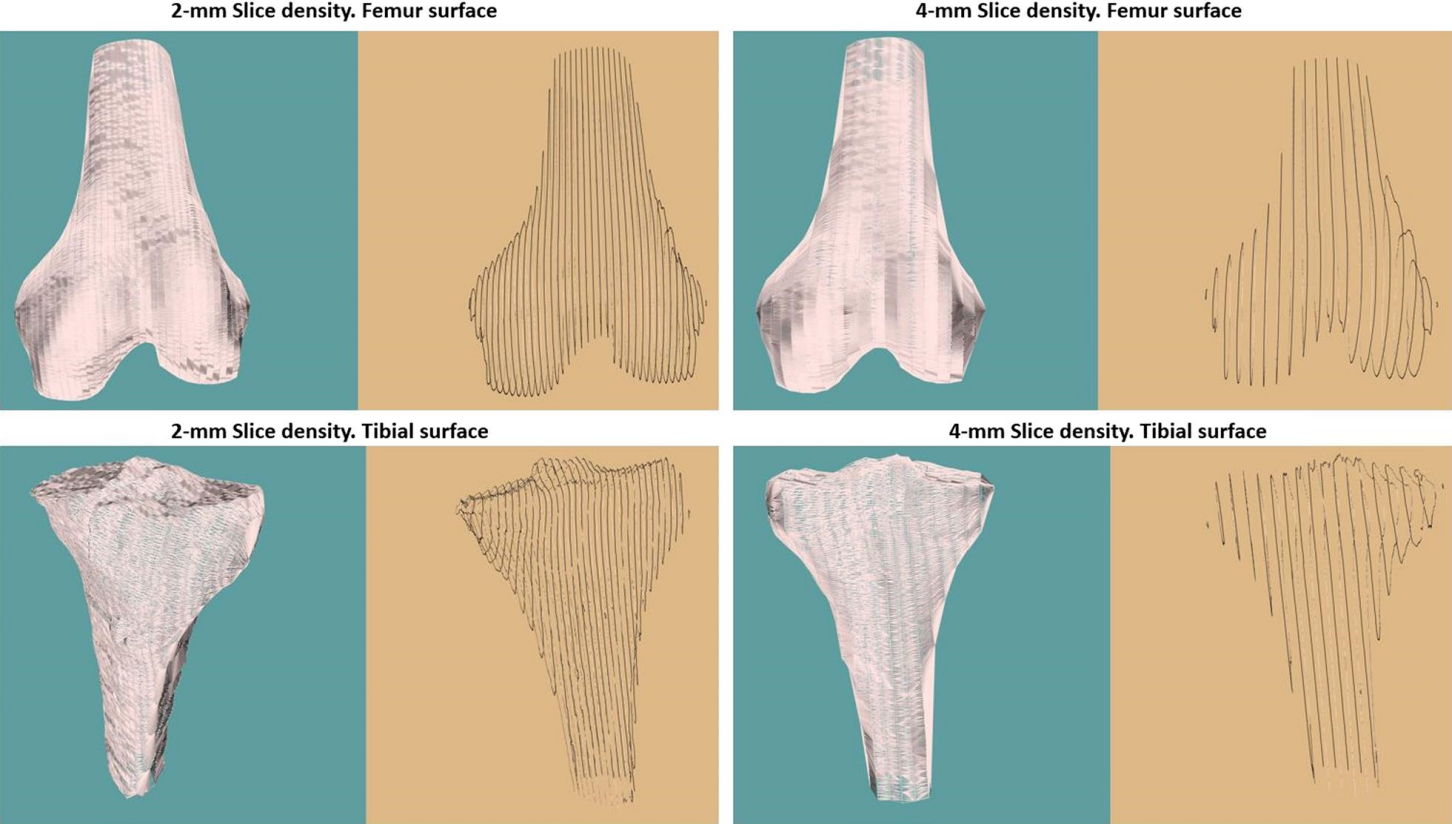
Surface reconstruction. 3D surface reconstruction of the femur and tibia for different slice densities

In this study, a set of Digital Imaging and Communications in Medicine (DICOM) images of femoral and tibial specimens were used. These images were used to generate 3D images of both these bones, and then, these 3D images were segmented. The segmented bones were stored as 2D contours contained in evenly spaced sagittal or axial images (slices). Then, the algorithm proposed in this paper was used to convert the 2D contours into 3D surfaces. Different spacing distances (from 1 to 5 mm) between the slices were used to test the results of the algorithm. The algorithm was developed in the programming language C++, and the VTK library was used to visualize the results. We chose to use qualitative analysis on the mesh since quantitative analysis was too complex for implementation in our timeframe. Several femur and tibia specimens were used to test the reconstruction ability of our algorithm. The algorithm was found to produce qualitatively good 3D meshes for all the set contours with different slice densities, as shown in the examples in Fig. 6. Some interesting observations were noted when there were rapid changes from one contour to the next; the contours were not similar in shape or size. These cases often caused some triangles on the mesh to converge toward a single point at the edge of the smaller contour. This can be a subjective issue, since the meshes are still qualitatively acceptable when rasterized.

## Discussion

Our results showed that our algorithm is capable of producing very good results for the 3D reconstruction of the surfaces of the femur and tibia. It is recommended that the algorithm be used with axial or sagittal slices to avoid the appearance of complex branches in the contours; these branches cannot be handled correctly by the algorithm unless the procedure chosen for branching is modified. Because there are different ways to achieve correct triangulation between two adjacent contours, there may be a degree of ambiguity with regard to the results of other reconstruction methods. When the two contours are similarly shaped, the ambiguity is negligible and the reasonable solutions will be similar. However, as the respective shapes of the serial sections become increasingly divergent, the ambiguity increases. To reduce this ambiguity, it is recommended that the contours be close enough, such that there is minimal variation between adjacent contour lines. This approach has the advantage of tending toward an exact description; however, in practice, such data may not be available. The algorithm tries to avoid this problem by performing a previous normalization of the contours. It is advisable to perform a quantitative study of the results obtained, since we have limited ourselves to only performing a qualitative analysis of the results, as well as improving the process of handling ramifications in the contours.

## Conclusions

In this work, a solution to the problem associated with the 3D reconstruction of femur and tibia models from a set of parallel and ordered sagittal or axial slices was proposed. The reconstruction proved to be quite effective using optimization based on minimizing the area of the triangles that form the 3D surface. In the tested examples, no significant improvements were shown when combining the optimization with a contour-normalization process, but as this study mentions, the use of normalization is recommended to avoid possible failures in the 3D reconstruction of more complex contours. The algorithm described herein can be used for the 3D reconstruction of other types of 2D cuts, but special attention must be paid with regard to the branches, because the proposed algorithm is designed for non-complex branching structures.

The problem of the correspondence between slices or looking for adjacent slices is not addressed in this study because this problem can be fixed keeping a control on the generated slices during segmentation to know the correct order between them. The use of coronal cuts is not recommended because these cuts can generate complex unconnected areas in adjacent sections that the algorithm cannot handle adequately.

There is an increased interest in computer-aided surgery in total knee arthroplasty [13] and in minimally invasive management of traumatic conditions around the knee [14, 15]. The opportunity to produce anatomically correct models preoperatively should help surgeons to better plan the procedures and improve outcomes.

## Data Availability

None.
